# *NGS-Logistics*: data infrastructure for efficient analysis of NGS sequence variants across multiple centers

**DOI:** 10.1186/1471-2105-16-S2-A10

**Published:** 2015-01-28

**Authors:** Amin Ardeshirdavani, Erika Souche, Luc Dehaspe, Jeroen Van Houdt, Joris Robert Vermeesch, Yves Moreau

**Affiliations:** 1 KU Leuven, Department of Electrical Engineering (ESAT), STADIUS Center for Dynamical Systems, Signal Processing and Data Analytics, Leuven, Belgium; 2 iMinds Medical IT Department. Kasteelpark Arenberg 10, Box 2446, 3001 Leuven, Belgium; 3 KU Leuven, Center of Human Genetics Gasthuisberg, O&N I Herestraat 49 - box 602, 3000 Leuven, Belgium; 4 KU Leuven Department of Human Genetics Gasthuisberg, O&N I Herestraat 49 - box 602, 3000 Leuven, Belgium

## Background

Next-Generation Sequencing (NGS) is a key tool in genomics, in particular in research and diagnostics of human Mendelian, oligogenic, and complex disorders [1]. Multiple projects now aim at mapping the human genetic variation on a large scale, such as the 1,000 Genomes Project, the UK 100k Genome Project. Meanwhile with the dramatic decrease of the price and turnaround time, large amounts of human sequencing data have been generated over the past decade [2]. As of January 2014, about 2,555 sequencers were spread over 920 centers across the world [3]. As a result, about 100,000 human exome have been sequenced so far [4]. Crucially, the speed at which NGS data is produced greatly surpasses Moore's law [5] and challenges our ability to conveniently store, exchange, and analyze this data. Data pre-processing is needed to extract reliable information from sequencing data and it can be divided into two major steps: primary analysis (image analysis and base calling) and secondary analysis. When looking for variation in the human genome, secondary analysis consists of aligning/mapping the reads against the reference genome and scanning the alignment for variation. Both raw data and mapped reads are large files occupying significant disk storage space. The collection of files resulting from the analysis of a single whole genome study can take up to 50Gb of disk space. This raises significant issues in terms of computing and data storage and transfer, with off-site data transfer currently being a key bottleneck. Moreover, the analysis of NGS data also raises the major challenge of how to reconcile federated analysis of personal genomic data and confidentiality of data to protect privacy. In many situations, the analysis of data from a single study alone will be much less powerful than if it can be correlated with other studies. In particular, when investigating a mutation of interest, it is extremely useful to obtain data about other patients or controls sharing similar mutations. However, personal genome data (whole genome, exome, transcriptome data, etc.) is sensitive personal data. Confidentiality of this data must be guaranteed at all times and only duly authorized researchers should access such personal data.

## Methods

**Figure 1:**
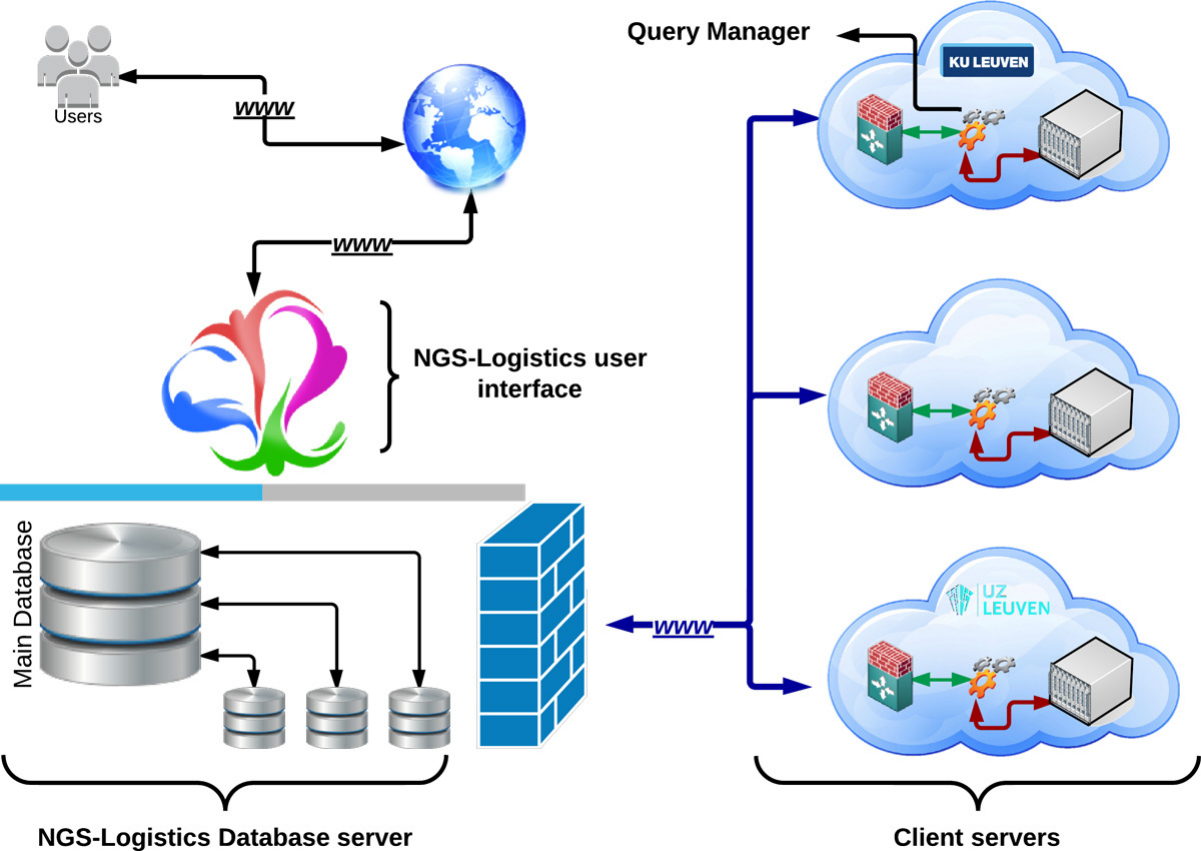
**NGS-Logistics components**. Users pass their queries from the NGS-Logistics web interface to the clients. Request are stored and scheduled in the main database. Each center has one database, being the only way of communication between centers and the main system. Centers and their databases are connected through a secured connection, to which only valid and trusted IPs are allowed to connect. The query manager is responsible for tracking and running the request, as well as collecting and returning the results to the main system.

**Figure 2:**
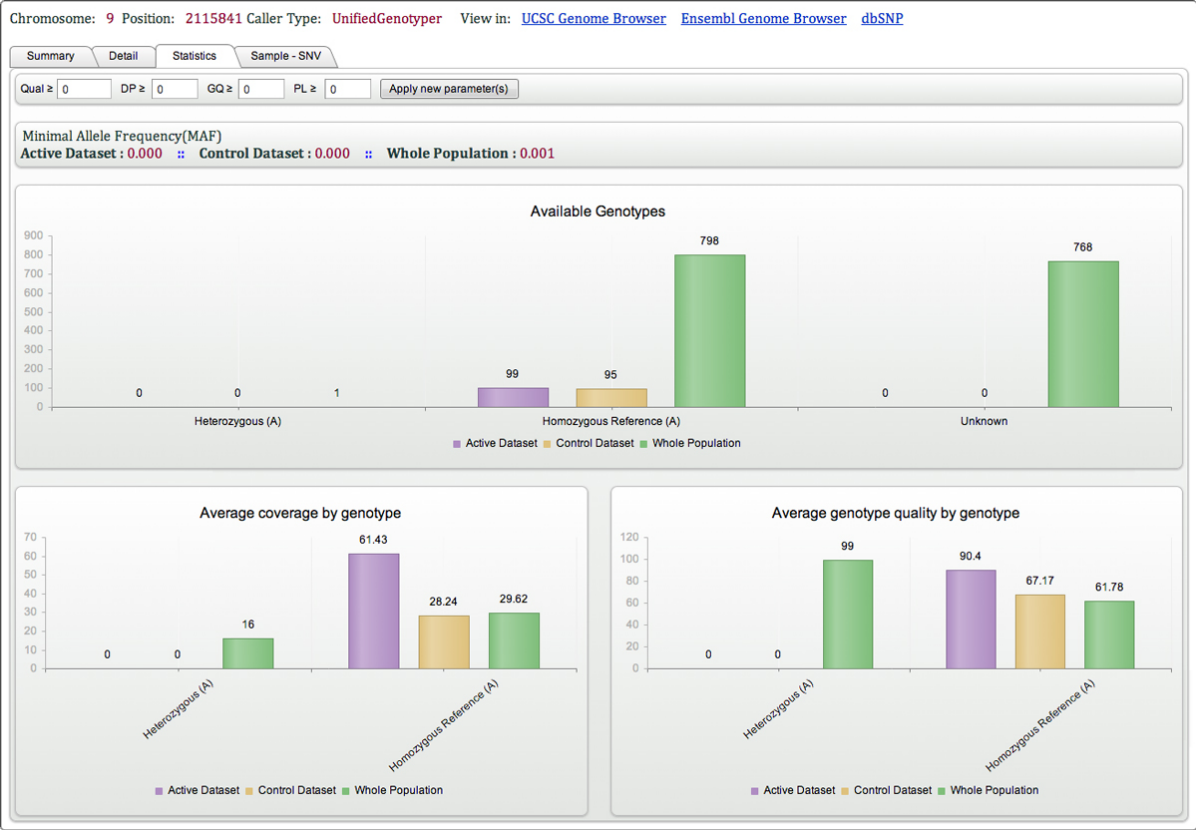
**Single Point Query result page (Statistic section) for chr9:2115841**. The query of chr9:2115841 shows that only one sample is polymorphic at this position. All samples that can be genotyped at this position from the active data set, control data set and whole are homozygous reference. The MAF of this variant in each data set is thus very low.

To address all challenges described above, we developed a data structure *NGS-Logistics*, which fulfills all requirements of a successful application that can process data inclusively and comprehensively from multiple sources while guaranteeing privacy and security. *NGS-Logistics *is a web-based application providing a data structure to analyze NGS data in a distributed way. The data can be located in any data center, anywhere in the world. *NGS-Logistics *provides an environment in which researchers do not need to worry about the physical location of the data (Figure 1). With respect to users rights, queries will be sent to each remote server. The host will process the request and return the results back to the main server where all the privacy limitations are controlled for the data. Once the results are ready, the end user can see the desired information. Depending on the type of query, results will be divided into two parts, the first part is related to the samples to which the user has authorized access, and for which the users can see all details. The second part contains results for the whole population, for which the user has only access to some aggregate statistics without details. An example of such a query would be to review the mutations present at a single genomic position in each individual patient from a set of patients to which the user has authorized access (1st part) and to contrast these results with background frequency of mutation in the reference populations (2nd part) (Figure 2).

## Results

The pilot version of *NGS-Logistics *has been installed and is currently being beta-tested by users at the Center for Human Genetics of the University of Leuven. Currently we have two installations of the system, the first one at the Leuven University Hospitals and the second one at the Flemish Supercomputing Center (VSC). The development of *NGS-Logistics *has significantly reduced the effort and time needed to evaluate the significance of mutations from full genome sequencing and exome sequencing, in a safe and confidential environment. This platform provides more opportunities for operators who are interested in expanding their queries and further analysis.

## References

[B1] Voelkerding KV Dames SA Durtschi JD Next-generation sequencing: from basic research to diagnostics Clin Chem 2009 55 4 641 658 10.1373/clinchem.2008.112789 19246620

[B2] Institute NHGR DNA Sequencing Costs 2013

[B3] Next Generation Genomics: World Map of High-throughput Sequencers http://omicsmaps.com/

[B4] Human genome: Genomes by the thousand Nature 2010 467 7319 1026 1027 20981067 10.1038/4671026a

[B5] DNA Sequencing Costs: Data from the NHGRI Genome Sequencing Program (GSP) http://www.genome.gov/sequencingcosts/

